# Utilization of anonymization techniques to create an external control arm for clinical trial data

**DOI:** 10.1186/s12874-023-02082-5

**Published:** 2023-11-04

**Authors:** Juha Mehtälä, Mehreen Ali, Timo Miettinen, Liisa Partanen, Kaisa Laapas, Petri T. Niemelä, Igor Khorlo, Sanna Ström, Samu Kurki, Jarno Vapalahti, Khaled Abdelgawwad, Jussi V. Leinonen

**Affiliations:** 1 MedEngine Oy, Helsinki, Finland; 2 Veil.ai Oy, Helsinki, Finland; 3 Institute for Molecular Medicine Finland (FIMM), HiLIFE, University of Helsinki, Helsinki, Finland; 4 Bayer Oy, Espoo, Finland; 5 Bayer AG, Berlin, Germany

**Keywords:** Anonymization, Real-world data, Randomized clinical trials, External control arm

## Abstract

**Background:**

Subject-level real-world data (RWD) collected during daily healthcare practices are increasingly used in medical research to assess questions that cannot be addressed in the context of a randomized controlled trial (RCT). A novel application of RWD arises from the need to create external control arms (ECAs) for single-arm RCTs. In the analysis of ECAs against RCT data, there is an evident need to manage and analyze RCT data and RWD in the same technical environment. In the Nordic countries, legal requirements may require that the original subject-level data be anonymized, i.e., modified so that the risk to identify any individual is minimal. The aim of this study was to conduct initial exploration on how well pseudonymized and anonymized RWD perform in the creation of an ECA for an RCT.

**Methods:**

This was a hybrid observational cohort study using clinical data from the control arm of the completed randomized phase II clinical trial (PACIFIC-AF) and RWD cohort from Finnish healthcare data sources. The initial pseudonymized RWD were anonymized within the (*k*, *ε*)-anonymity framework (a model for protecting individuals against identification). Propensity score matching and weighting methods were applied to the anonymized and pseudonymized RWD, to balance potential confounders against the RCT data. Descriptive statistics for the potential confounders and overall survival analyses were conducted prior to and after matching and weighting, using both the pseudonymized and anonymized RWD sets.

**Results:**

Anonymization affected the baseline characteristics of potential confounders only marginally. The greatest difference was in the prevalence of chronic obstructive pulmonary disease (4.6% vs. 5.4% in the pseudonymized compared to the anonymized data, respectively). Moreover, the overall survival changed in anonymization by only 8% (95% CI 4–22%). Both the pseudonymized and anonymized RWD were able to produce matched ECAs for the RCT data. Anonymization after matching impacted overall survival analysis by 22% (95% CI -21–87%).

**Conclusions:**

Anonymization may be a viable technique for cases where flexible data transfer and sharing are required. As anonymization necessarily affects some aspects of the original data, further research and careful consideration of anonymization strategies are needed.

**Supplementary Information:**

The online version contains supplementary material available at 10.1186/s12874-023-02082-5.

## Introduction

Real-world data (RWD) collected during daily healthcare practices are increasingly used in medical research to assess questions that cannot be addressed in the context of randomized controlled trials (RCT) [1–3]. Some of the most common applications of RWD are studies on the effectiveness and safety of medical products in real-life clinical practice, evaluation of disease epidemiology and economic burden, as well as support in drug development, clinical trial design, product marketing, and regulatory approval [3–7].

A novel application of RWD rises from the need to create external control arms (ECAs) for single-arm RCTs [8]. In such an application, RWD sources are utilized to create a comparator that mimics the characteristics of an RCT arm. This is especially relevant when a novel treatment has been shown to be highly efficacious, the disease in question is rare or very serious, when no effective standard treatments are available, or the target populations are too small [9, 10]. In such cases, ethical considerations or infeasibility may not support regular double-blinded placebo-controlled RCTs [11–15]. Moreover, by reducing or eliminating the need to enroll control patients for two RCT arms, an ECA can also increase efficiency, reduce delays, and lower costs in the evaluation of new therapies. In the creation of an ECA using RWD, challenges may arise due to differences in data availability and quality. These aspects may affect the semantic and syntactical interoperability, and hinder acquisition of similar cohort characteristics.

In the Nordic countries, including Finland, Sweden, Norway, and Denmark, comprehensive healthcare data are recorded in an electronic format in national healthcare registers, providing an excellent ecosystem to utilize RWD [16]. Within the Nordics, pseudonymized individual-level RWD may be used for research purposes by applying for a research permit [17–19]. Utilizing RWD in the Nordics for the creation of an ECA and analysis against RCT data implies that the data analysis is done in the same technical environment (e.g., computing infrastructure designed for securing sensitive data), and RWD are pseudonymized (direct identifiers such as name or social security number are removed) before being available for analysis in the secure environment [19]. When pseudonymized RWD are extracted from the secure environment, the data needs to be anonymized (the risk to identify an individual even indirectly is minimized) [19]. This is due to tight laws and regulations on data protection since the protection of individual-level data is considered a top priority in the EU [20].

In the Nordics, there are essentially two options to analyze RWD against RCT data. In the first option, RCT data are transferred to the secure environment where the pseudonymized RWD are located, and access to that environment is granted to all relevant parties. In the second option, anonymized RWD are extracted into the technical environment in which the RCT data are located. The feasibility of the first option depends on regulations that govern the transfer of RCT data, while the feasibility of the second option depends on the amount of information lost in the anonymization process of the originally pseudonymized RWD.

A well-established and commonly accepted definition of anonymized data is not available. Recital 26 of the GDPR defines requirements for anonymization to be considered safe and acceptable [21]. For data to be considered anonymous under the GDPR, the anonymization procedure should ensure that re-identification is no longer possible using reasonable means, which are defined by, such as how much it would cost or how long it would take to identify someone from the data, what other relevant resources the users of the data would have access to, and what technology would be used now or in the foreseeable future. While the definition of Recital 26 establishes reasonable principles for anonymization, implementing these requirements in real-world use cases and datasets, given the capabilities and limitations of the technology, can be challenging [22].

Anonymization depends on how it is defined and, consequently, what types of anonymization techniques are utilized. Anonymization techniques include, but are not limited to, micro aggregation, noise addition, rank swapping, shuffling, recoding, and local suppression [23]. Several measures for the estimation of risk to identify individuals have been proposed [23], but regional agencies that govern the data may have contradictory interpretations. The selection of anonymization techniques and privacy criteria depends on the scope of the target data, variable types, and the intended use of the resulting anonymized data. Some of the recent examples of publishing anonymized RWD include the Lean European Open Survey on SARS-CoV-2 Infected Patients (LEOSS), [24, 25] European population statistics, [26] and urban mobility data [27]. There are also studies analyzing the privacy risks and data accuracy trade-offs of anonymized RWD and clinical study data sets [26–30].

The aim of this study was to explore how well anonymized RWD performs in the creation of an ECA for an RCT, when compared to the corresponding performance prior to the anonymization, i.e., when using pseudonymized RWD. Furthermore, the study compared general characteristics of the same pseudonymized and anonymized RWD sets, to assess the magnitude of discrepancies caused by anonymization. The study was based on one RCT and one RWD, and the scope of this study was the establishment of concepts, and investigation of their utility in one use case.

## Materials and methods

### Study design, setting and participants

This was a hybrid observational cohort study using clinical data from the control arm of the completed randomized phase II clinical trial (PACIFIC-AF) and a RWD from Finnish healthcare data sources. The RCT included a total 755 patients with atrial fibrillation (AF) in three arms, and was originally designed to investigate a new factor XIa inhibitor, and to compare its safety and efficacy against an existing oral anticoagulant (apixaban) [31, 32]. This study was conducted independently and is a post hoc analysis of the original RCT. To imitate data gathering as in a single arm RCT, the apixaban arm of 250 patients was utilized. The RWD were collected using selection criteria that followed the RCT design as applicable. The specific inclusion criteria were 1) age ≥ 45; 2) prescription and usage of novel oral anticoagulant (NOAC) medication (Anatomical Therapeutic Chemical classification system [ATC] rivaroxaban [ATC: B01AF01], apixaban [ATC: B01AF02], edoxaban [ATC: B01AF03], or dabigatran [ATC: B01AE07] between 1^st^ January 2014 and 30^th^ September 2019; and 3) patients who were diagnosed with atrial fibrillation (AF) (international classification of diseases, 10^th^ revision [ICD-10] I48 or ICD, 9^th^ revision [ICD-9] 4273A) prior to the NOAC initiation. The full RWD cohort was identified from Auria Data Lake by the Hospital District of Southwest Finland. A total of 8,255 patients fulfilled the selection criteria. To further mimic the RWD design with the RCT, more specific selection criteria were applied, resulting in selection of 3,327 patients of the total possible 8,255. The RWD resulted from a non-interventional, retrospective study that did not affect the physicians’ management of the patients.

To ensure a similar proportion of NOAC-naïve patients in the RWD cohort vs. current apixaban-using patients at the RCT study entry, an algorithm that transforms a portion of the patients into current users at study entry was applied. For these artificial current users, the date of study cohort entry was defined as an “artificial index date” based on the observed time on NOAC treatment in the RCT. For NOAC-naïve patients, the study entry date was defined as the date of first NOAC use. Data prior to the study entry date was considered as baseline data, and patients were followed-up from study entry until death, and maximally up to 31^st^ December 2020.

### Variables

Thirty-six variables (explained in detail in the Supplementary Information file, see Supplementary Table 1) that were considered as potential confounders were included as baseline data. The investigated outcome was overall survival, defined as time from study entry to death event or censoring at 31^st^ December 2020, whichever occurred first. Both RWD and RCT included the baseline data, while only RWD included the outcome.

### Data sources

The study data were collected from a primary data source (PACIFIC-AF RCT data) and secondary data sources (RWD)*.* The RCT primary data collection source was the PACIFIC-AF phase II clinical trial (ClinicalTrials.gov Identifier: NCT04218266), and baseline data (without outcomes) for patients using apixaban for AF were included [31, 32]. The RWD were collected from both the regional hospital data lake of Southwest Finland (via Auria Clinical Informatics), and the following national Finnish healthcare registries: the nationwide prescription registers—Prescription Centre and Drug Prescription Registry by the Social Insurance Institution of Finland (Kela); the nationwide healthcare registers—Care Register for Health care, and the Register of Primary Health Care Visits by the Finnish Institute for Health and Welfare (THL); and the nationwide cause of death register by Statistics Finland.

### Pseudonymized and anonymized data

The following outlines the methodological selection of the anonymization framework, and more detailed information is given in the supplementary information. The data authority that regulates the use of RWD in Finland (Findata) requires the use of *k*-anonymity (*k* = 5), and this was considered as the starting point for the selection of the anonymization framework. Given this premise, two candidate approaches were evaluated using membership inference attacks [33–35]. First, the ε-safe k-anonymization [34] that may offer slight improvement against membership inference attacks, and it also considers the differential privacy composability problem in the case of multiple data publications. Second, the (*k, ε*)-anonymity framework [33] that was eventually selected, as the ε-safe k-anonymization method [34] is based on sampling, and data quality is more difficult to control, especially with imbalanced data.

The driving factor for the selection of *ε* value for this project was to ensure that anonymized real-world data had adequate privacy protections for it to be considered safe by the data authority (Findata). According to [36] the maximum privacy risk for the study dataset for *ε* values 0.01 to 7 results in $\rho$ = 0.012%–11.77%. A value *ε* = 3.46 (ln 32), from the middle of the range of recommended values, yields a maximum privacy risk $\rho$ = 0.39%. This was selected as the initial *ε* value, for which privacy was evaluated by using the membership inference attack [35].

The pseudonymized data sets included subject-level data on all RWD and RCT study participants, without direct identifiers such as name or social security number. The anonymized RWD were derived from the pseudonymized RWD with *k*-anonymity criteria (*k* = 5) for all equivalence classes of size *k,* defined by quasi-identifying variables and *ε*-differential privacy criteria (*ε* = 3.46) for all non-quasi-identifying variables [33, 37]. The RWD were transformed according to the variable type and privacy criteria (Table 1, Supplementary Table 1). The exponential differential privacy mechanism was applied to categorical variables, including quasi-identifying variables, and to the records that failed the k-criteria, whereas the Laplace mechanism was used for numerical variables [34, 38, 39]. The data were then cleaned of nonsensical and out-of-range values produced by the differential privacy mechanism. Finally, the order of anonymized records was shuffled and the subject identifiers were replaced by random record identifiers. The number of records in the anonymized RWD is unaffected by these transformations. The Finnish data authority (Findata) approved the level of anonymization with the selected anonymization framework.

**Table 1:** Summary of transformations and privacy criteria used by variable type

**Variable types**	**Privacy Criteria**	**Transformations** *(Anonymization method)*
Identifiers		Suppression
Quasi identifiers	(*k*, ε)—anonymity	Noise (exponential mechanism) Sampling^a^
Numerical variables	Differential privacy	Noise (Laplace mechanism)
Categorical variables	Differential privacy	Noise (exponential mechanism)
Metadata (record order within a table)	Record order-based attacks	Shuffling

^a^Sampling was conducted as part of the exponential differential privacy mechanism and specific for the records that failed the *k*-criteria

### Statistical analyses

The logistic-regression model in which all 36 potential confounders were involved was used to estimate the propensity score (PS) for being in the RCT arm. In matching, the logit of the PS was used with caliper matching (width equal to 0.2) at the artificial index date [40]. In matching weighting (MW), pairwise algorithmic matching was used. Matching weight was defined as the smaller of the predicted probabilities of receiving or not receiving the treatment over the predicted probability of being assigned to the arm where the patient is [41]. In addition, the PS overlap weighting (OW) method was utilized [42]. After matching and weighting, standardized mean differences (SMDs) below 0.1 were considered as success and values between 0.1–0.25 as moderate success [43].

For all included patients, descriptive statistics were presented separately prior to and after matching and weighting, using both anonymized and pseudonymized data. Continuous variables were described by mean, standard deviation (SD), median, 25^th^, and 75^th^ percentiles. Categorical variables were described by proportion and frequency in each category.

The Kaplan–Meier method was used to assess the time-to-event outcome prior to and after matching, using both the pseudonymized and anonymized data [44]. In addition, the Cox regression method was used to assess the association between the outcome and the confounders in the pseudonymized and anonymized data sets prior to matching [45].

## Results

### Analyses prior to matching

Baseline description of the 3,327 patients included in the pseudonymized and anonymized RWD, and for the 250 patients included in the RCT, is given in Table 2. In the full RWD, the results show that anonymization affects the population mean and proportion statistics only minimally. The greatest SMD between the pseudonymized and anonymized RWD sets is for chronic obstructive pulmonary disease (COPD), which was present in 4.6% (152/3,327) in the pseudonymized data and 5.4% (181/3,327) in the anonymized data (SMD = 0.04 for COPD, and SMD < 0.04 for all other variables, values not shown).

**Table 2:** Baseline descriptions of the pseudonymized and anonymized real-world data and randomized controlled trial data sets

Variable	Pseudonymized	Anonymized	RCT
	**RWD**	**RWD**	
N	3,327	3,327	250
Age, mean (SD)	75.92 (9.19)	75.84 (10.22)	74.27 (8.32)
Anemia, n (%)	761 (22.9)	770 (23.1)	26 (10.4)
Anti-diabetic medication use, n (%)	954 (28.7)	963 (28.9)	76 (30.4)
Anti-hypertensive medication use, n (%)	3,228 (97.0)	3,229 (97.1)	247 (98.8)
Aortic arteriosclerosis, n (%)	< 5 (< 0.2)	< 5 (< 0.2)	< 5 (< 2.0)
Arterial hypertension, n (%)	2,226 (66.9)	2,223 (66.8)	220 (88.0)
BMI ≥ 30 kg/m^2^, n (%)	470 (14.1)	485 (14.6)	84 (33.6)
Carotid endarterectomy or stent, n (%)	9 (0.3)	9 (0.3)	< 5 (< 2.0)
Chronic heart failure, n (%)	696 (20.9)	710 (21.3)	117 (46.8)
Chronic kidney disease, n (%)	477 (14.3)	477 (14.3)	41 (16.4)
COPD, n (%)	152 (4.6)	181 (5.4)	24 (9.6)
Coronary artery disease, n (%)	709 (21.3)	729 (21.9)	50 (20.0)
Diabetes mellitus, n (%)	832 (25.0)	832 (25.0)	87 (34.8)
History of ISTH major bleeding, n (%)	137 (4.1)	149 (4.5)	22 (8.8)
History of osteoporotic fracture, n (%)	165 (5.0)	175 (5.3)	5 (2.0)
History of stroke, n (%)	305 (9.2)	313 (9.4)	20 (8.0)
Hyperlipidemia, n (%)	565 (17.0)	579 (17.4)	92 (36.8)
Hyperthyroidism, n (%)	70 (2.1)	72 (2.2)	< 5 (< 2.0)
Hypothyroidism, n (%)	530 (15.9)	543 (16.3)	28 (11.2)
Low body weight (body weight < 60 kg), n (%)	3,091 (92.9)	3,063 (92.1)	221 (88.4)
Malignancy, n (%)	441 (13.3)	442 (13.3)	45 (18.0)
Myocardial infarction, n (%)	161 (4.8)	165 (5.0)	36 (14.4)
Non-steroidal anti-inflammatory drugs, n (%)	825 (24.8)	833 (25.0)	18 (7.2)
Percutaneous coronary intervention, n (%)	202 (6.1)	203 (6.1)	15 (6.0)
Peripheral arterial disease, n (%)	113 (3.4)	118 (3.5)	20 (8.0)
Platelet aggregation inhibitors, n (%)	3,023 (90.9)	3,018 (90.7)	234 (93.6)
Prior or concomitant use of Histamine-2, n (%)	18 (0.5)	18 (0.5)	< 5 (< 2.0)
Prior or concomitant use of SSRIs, n (%)	176 (5.3)	179 (5.4)	6 (2.4)
Prior use of heparins, n (%)	1,059 (31.8)	1,081 (32.5)	55 (22.0)
Prior use of NOACs, n (%)	1,936 (58.2)	1,927 (57.9)	146 (58.4)
Serum creatinine ≥ 1.5 mg/dL, n (%)	392 (11.8)	399 (12.0)	27 (10.8)
Sex, Male, n (%)	1,622 (48.8)	1,616 (48.6)	141 (56.4)
Smoking status, n (%)	273 (8.2)	303 (9.1)	10 (4.0)
TIA, n (%)	111 (3.3)	113 (3.4)	13 (5.2)
Time in days since atrial fibrillation (%)			
≤ *30*	693 (20.8)	696 (20.9)	99 (39.6)
> 30 – < 90	2,414 (72.6)	2,415 (72.6)	135 (54.0)
≥ 90	220 (6.6)	216 (6.5)	16 (6.4)
Use of proton pump inhibitors, n (%)	1,494 (44.9)	1,503 (45.2)	109 (43.6)

*Abbreviations*: *BMI* Body mass index, *COPD* Chronic obstructive pulmonary disease, *ISTH* International Society on Thrombosis and Haemostasis, *NOAC* Novel oral anticoagulant, *RCT* Randomized controlled trial, *RWD* Real-world data, *SD* Standard deviation; SSRI, selective serotonin reuptake inhibitors, *TIA* Transient ischemic attack

For nearly all the variables presented in Table 2 there is a marked difference in the mean (continuous variables) or proportion (categorical variables) between the RWD and RCT sets, regardless of whether RWD is anonymized or pseudonymized. This indicates that the applied inclusion and exclusion criteria are not sufficient to harmonize these populations, and further covariate balancing by matching or weighting is required.

**Fig. 1:**
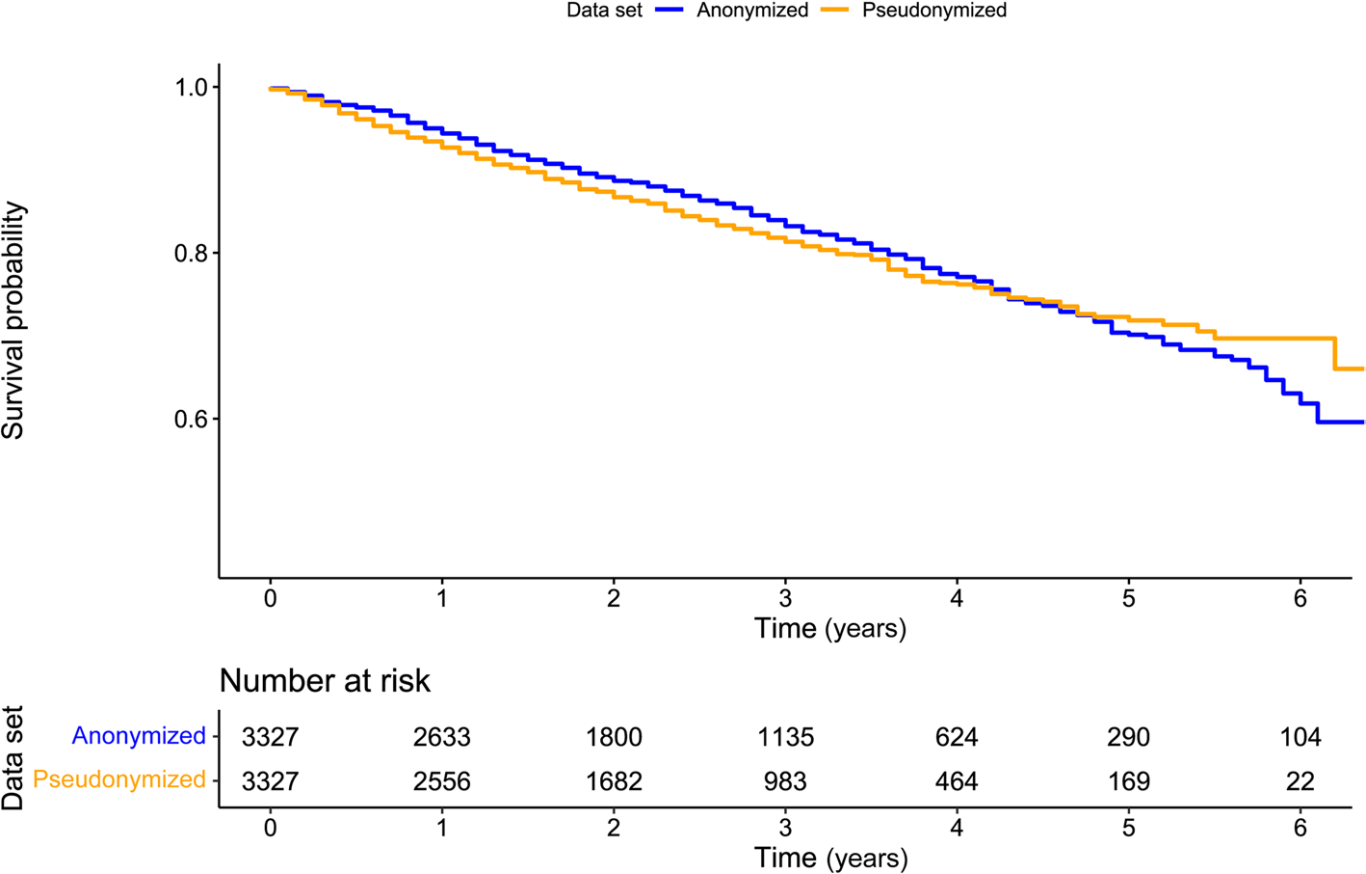
Kaplan–Meier estimates for overall survival from study entry in anonymized and pseudonymized real-world data sets

The overall survival for pseudonymized vs. anonymized data prior to matching, estimated using the Kaplan–Meier method, is given in Fig. 1. When measured using the Cox model, anonymization increased the overall survival, on average, by 8%: hazard ratio (HR) = 1.08 and 95% confidence interval (CI) = 0.96–1.22, *p* = 0.204 However, the difference is not statistically significant.

**Fig. 2:**
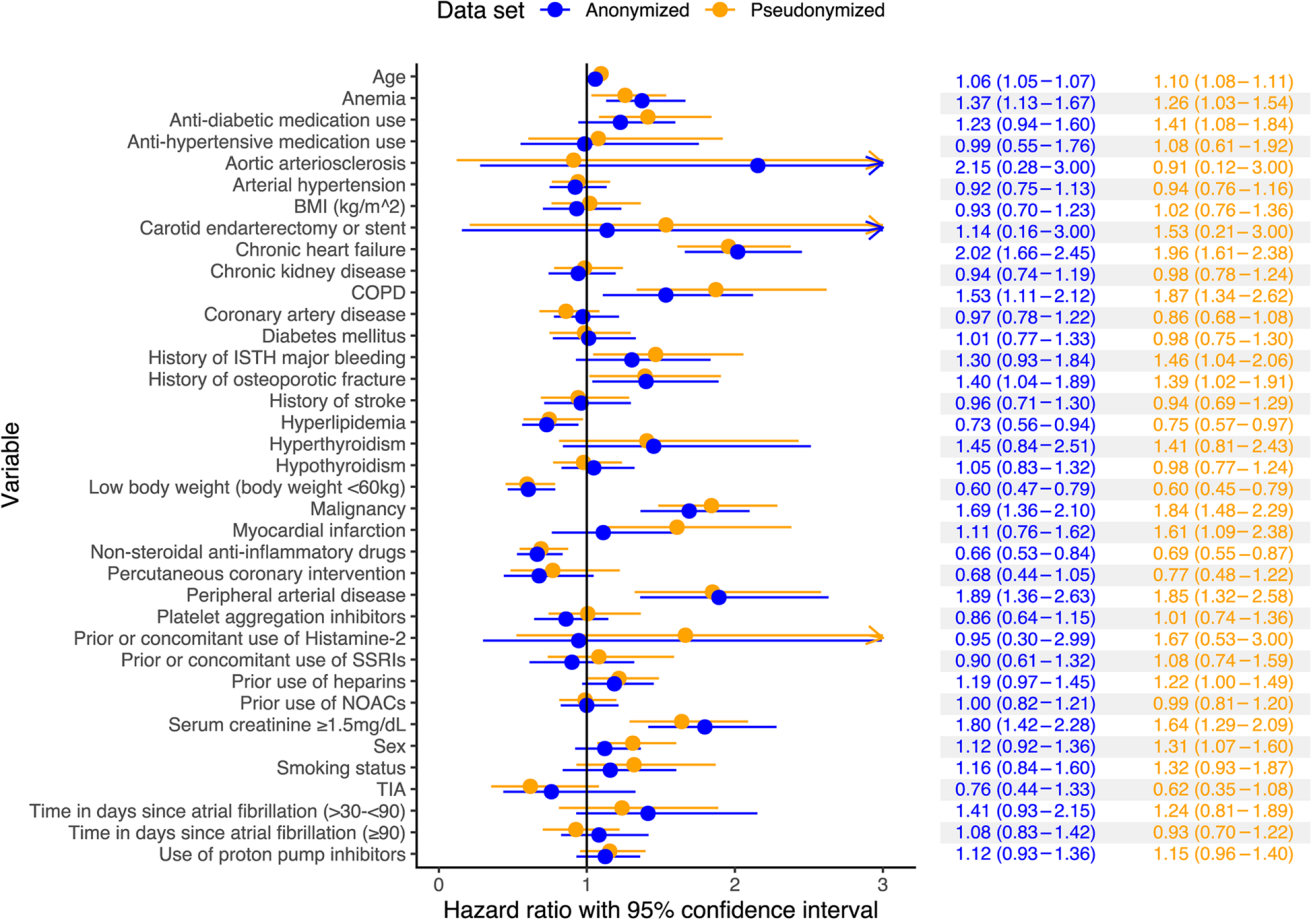
Cox model results for the association between overall survival and the confounders in fully anonymized and pseudonymized real world data sets. BMI, body-mass index (kg/m^2^); COPD, chronic obstructive pulmonary disease; NOAC, novel oral anticoagulant; SSRI, selective serotonin reuptake inhibitor; TIA, transient ischemic attack

The association between the 36 confounders and overall survival in the full pseudonymized and anonymized RWD sets is presented in Fig. 2. The greatest differences in the estimated hazard ratios were observed for aortic arteriosclerosis (the absolute difference in point estimates was equal to 1.24), prior or concomitant use of histamine-2 (0.72), and myocardial infarction (0.50), carotid endarterectomy or stent (0.39), and COPD (0.34), all of which had wide CIs. On the contrary, for peripheral arterial disease and hyperthyroidism that had wide CIs, anonymization affected the point estimates less than 0.05 units. The association between confounders and allocation to the RCT group (PS-model effects) using the pseudonymized and full anonymized RWD sets are shown in the Supplementary Information file, see Supplementary Fig. 1.

### Matching the RWD sets with the RCT data

**Fig. 3:**
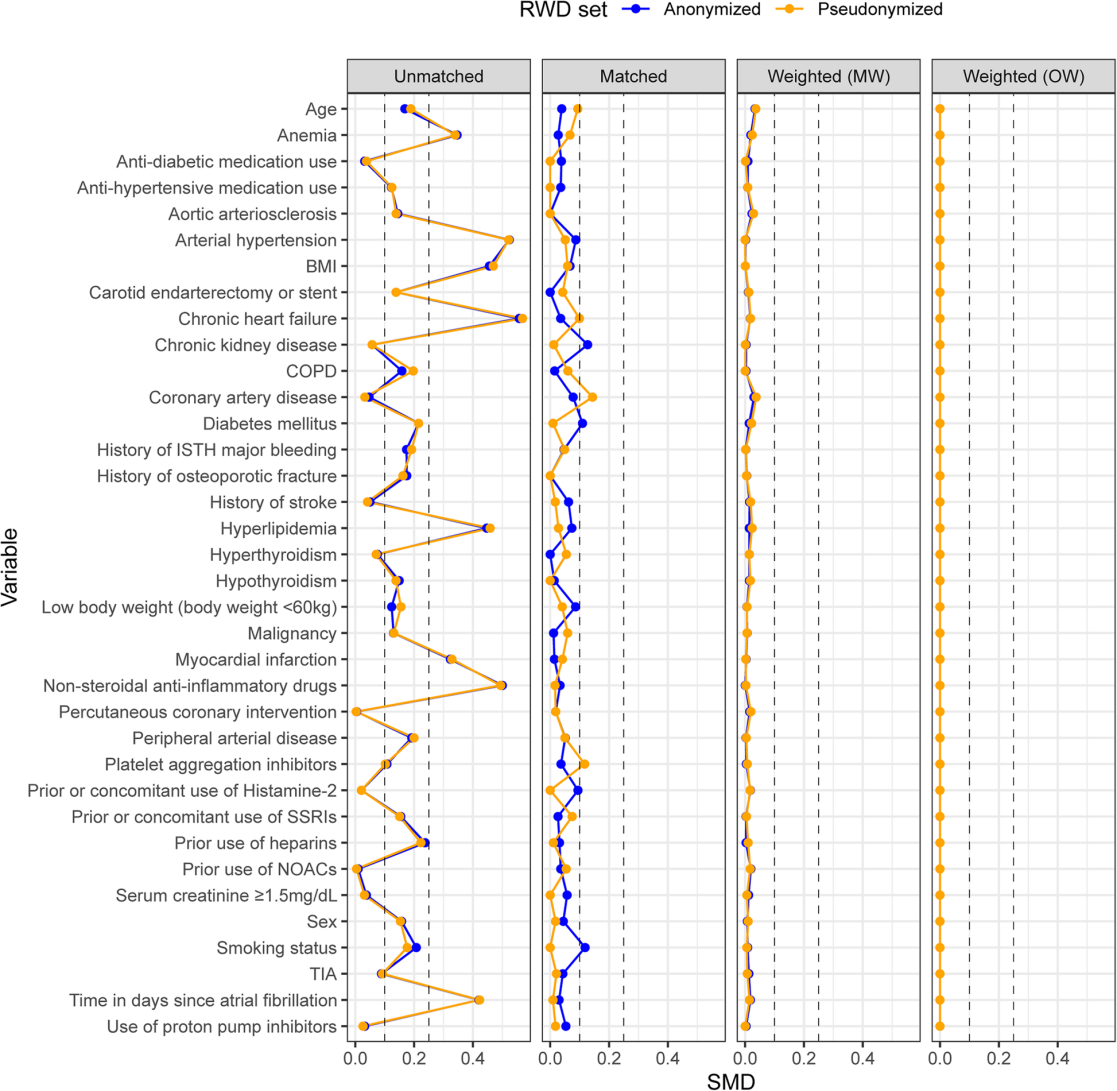
Standardized mean differences for the pseudonymized and anonymized real-world and randomized clinical trial data sets. Standardized mean differences are shown for prior to matching, after matching, after matching weighting, and after overlap weighting groups. Values for the anonymized set that are not visible are approximately equal to the pseudonymized ones. BMI, body-mass index (kg/m^2^); COPD, chronic obstructive pulmonary disease; MW, matching weighting; NOAC, novel oral anticoagulant; OW, overlap weighting; RWD, real-world data; SMD, standardized mean difference; SSRI, selective serotonin reuptake inhibitor; TIA, transient ischemic attack

Using both the pseudonymized and anonymized data (Fig. 3), matching the RWD to the RCT data was applicable, so that all the variables had SMD below 0.25, and only some were above 0.1. In the pseudonymized data, 2 out of the 36 variables (coronary artery disease and platelet aggregation inhibitors) had SMD > 0.1. With anonymized data, the corresponding variables (3/36) were chronic kidney disease, diabetes mellitus and smoking status. With both data sets, approximately the same number of matches was found: 223 with pseudonymized and 226 with anonymized (in more detail in the Supplementary Information file, see Supplementary Table 2). When using the weighting methods, the number of effective patients dropped (MW to 220–222, OW to 174–175), and the SMDs became smaller than 0.04.

### Comparison of the outcome after matching

**Fig. 4:**
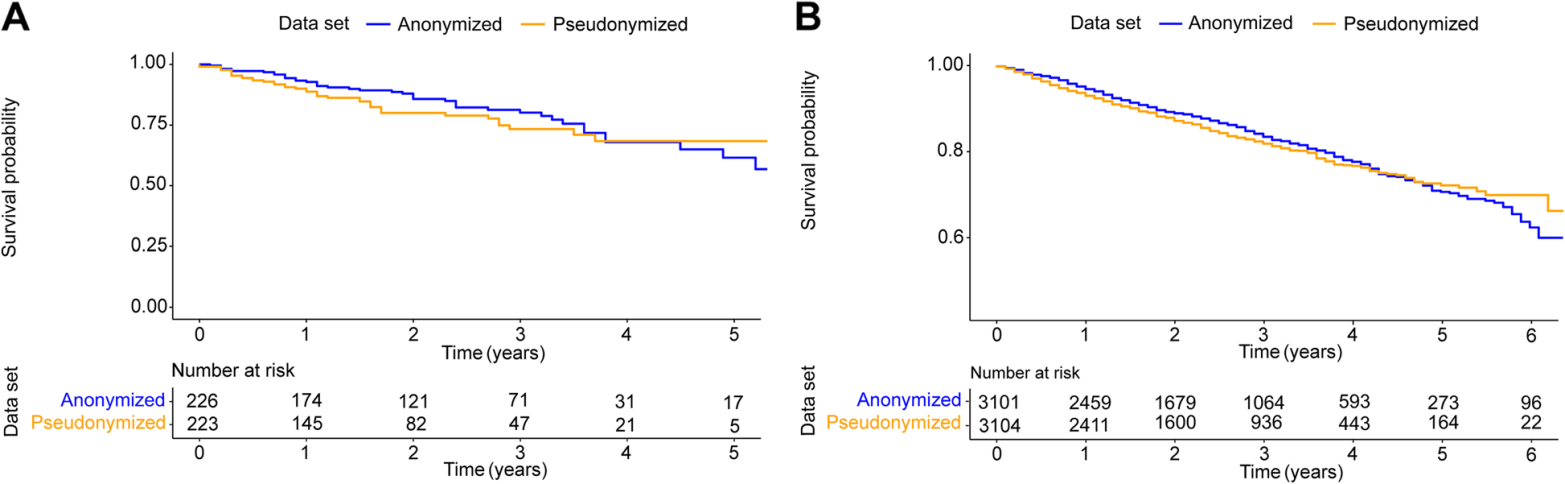
Overall survival estimates for pseudonymized and anonymized data sets. **A** Kaplan–Meier estimates for overall survival from study entry in matched pseudonymized and anonymized real-world data sets and (**B**) Kaplan–Meier estimates for overall survival from study entry in non-matched (all except matched) pseudonymized and anonymized real world data sets

The change in overall survival for matched pseudonymized vs. anonymized data estimated using the Kaplan–Meier method is given in Fig. 4A, and the corresponding result for non-matched (all except matched) in Fig. 4B. For matched data, the estimated hazard ratio indicated that anonymization changed the analysis of the outcome by 22% (HR = 1.22, 95%; CI = 0.79–1.87; *p* = 0.369), and for non-matched (all except matched), by 8% (HR = 1.08, 95%; CI = 0.94–1.22; *p* = 0.257).

## Discussion

This study investigated the utility of anonymized data in the context of creation of an external RWD control arm for an RCT. First, anonymization affected the baseline characteristics marginally, and the greatest difference was observed in the prevalence of COPD (4.6% in the pseudonymized data vs. 5.4% in the anonymized data). In addition, the overall survival changed by 8% (95% CI 4–22%) after anonymization. Second, both the pseudonymized and anonymized RWD were able to produce matched ECAs for the RCT data. Anonymization impacted the analysis of overall survival after matching by 22% (95% CI -21–87%). As the baseline characteristics after matching were nearly equal in both data sets, it is important to determine the cause in the observed difference in overall survival.

In a sensitivity analysis constructed to explore this observation, only the baseline covariates, and not the overall survival was anonymized. In this test, the overall survival in the matched population was impacted by nearly the same amount (22%) by anonymization. Since in this analysis the only distinction was selection of patients through matching, this result seems to indicate that different patients were matched to the RCT data when using the pseudonymized and anonymized RWD sets.

Regarding the other main findings, the distribution of overall survival prior to matching, baseline variable distributions, and PS-matching statistics were impacted relatively little by anonymization. In contrast, for several variables with wide CIs, the association between the baseline covariates and the outcome was markedly affected by anonymization. These results seem to indicate that anonymization has a larger impact on the results when estimation is dependent on individual data points, and less so when results are dependent on larger-scale population statistics. These findings give high-level insights into cases where a specific anonymization strategy may or may not be feasible. The quality of the resulting anonymized data depends on the algorithm used and the variables prioritized for matching. Therefore, it is crucial to consider case-specific requirements and privacy criteria when designing the anonymization strategy for the data. This study examined only one use case, due to the vast scope needed for achieving generalizable results. Indeed, as all RCTs have their own specific characteristics which need to be matched with RWD, and data holders may have different interpretations about legal aspects for privacy criteria, future work is needed to achieve more general quantitative recommendations about the applicability of different anonymization frameworks.

This was only one case-study in which anonymization of data might be of interest. Due to the regulatory requirements of clinical trials, creating an ECA is in the highest-end when it comes to the need to pertain to data usability. Creating an external control arm is already challenging due to the complexity of data harmonization and high regulatory requirements; adding anonymization to the process, as in this case-example, further complicates it. The intrinsic uncertainty and noise added by anonymization may be incompatible with some downstream analyses, such as the matching algorithms. Therefore, for such studies, the current recommendation is to make data transfer of pseudonymized RWD or RCT data permissible. However, when the analyses rely on population-level distributions, and less on individual data points, anonymization seems to perform particularly well.

The main focus in this paper was to demonstrate how anonymization affects the performance of RWD in the creation of an ECA in one use case. It was also assessed how well variables in the RWD and RCT sets reflect the same entity (data validation), and how well variables were selected for the PS-model to minimize any residual confounding. Due to the added complexity of such challenges in the creation of an ECA, it is also recommended to reduce any avoidable complexities. In the Nordics, this means that the RCT would be optimally transferred to the secure environment in which the RWD reside, to preserve the pseudonymized data as it comes from the registers and retain the maximum amount of information. This may require careful considerations and regulatory preparations early in the planning phase of such a study.

It is to be noted that the creation of an ECA includes several other factors that pose possibly serious challenges. First, the primary purpose of RWD is to support daily healthcare practices, and research is often referred to as secondary use of these data [17]. Thus, the quality of RWD recorded in daily healthcare depends heavily on the data collection practices [8, 46, 47]. In contrast, RCT data are referred to as primary data, since they are collected in the course of original research within a particular study, and quality of the resulting data is generally high. Second, due to lack of randomization in RWD, treatment allocation is not independent of patients’ history [48]. While these challenges are important in their own right, they were not assessed in this study.

Finally, for studies that may depend on small samples and individual data points, careful consideration of anonymization and data-analysis strategy should be made. When applied to cases that rely on large-scale population statistics, the benefits of anonymization may be substantial, when considered against the relatively marginal limitations. Even if anonymization may not be an optimal solution for all cases, our study shows that it can be a viable option when flexible data transfer and sharing is required.

### Supplementary Information

**Additional file 1: Table 1. **Definitions of potential confounders. **Table 2.** Baseline summaries for the pseudonymized and anonymized real-world data sets and randomized controlled trial data set after propensity score matching, after PS-matching weighting, and after PS-overlap weighting. **Figure 1. **Logistic regression model results (propensity-score model effects) for the confounders in anonymized and pseudonymized real-world data sets.

## Data Availability

Regarding the RWD, according to the Finnish legislation, access to individual-level data is restricted only to individuals named in the study permit. The study protocol is available upon request from the corresponding author. Regarding the RCT data, the data are not publicly available due to containing information that could compromise research participant privacy/consent.

## Abbreviations list

AF: Atrial fibrillation
ATC: Anatomical Therapeutic Chemical classification system
BMI: Body mass index
CI: Confidence interval
COPD: Chronic obstructive pulmonary disease
ECA: External control arm
eGFR: Estimated glomerular filtration rate
GPP: Good Pharmacoepidemiology Practice
GDPR: General Data Protection Regulation
Hb: Hemoglobin
HR: Hazard ratio
ICD-9: International classification of diseases, 9^th^ revision
ICD-10: International classification of diseases, 10^th^ revision
ICPC-2: International Classification of Primary Care, 2^nd^ edition
ISTH: International Society on Thrombosis and Haemostasis
MW: Matching weighing
NCSP: NOMESCO Classification of Surgical Procedures
NOAC: Novel oral anticoagulant
NSAID: Non-steroidal anti-inflammatory drugs
OW: Overlap weighing
PS: Propensity score
RCT: Randomized control trials
RWD: Real-world Data
SD: Standard deviation
SMD: Standardized mean differences
SSRI: Selective serotonin reuptake inhibitors
TIA: Transient ischemic attack
THL: Finnish Institute for Health and Welfare
