# Supplementary material for: Utilization of anonymization techniques to create an external control arm for clinical trial data
Source: BMC Med Res Methodol. 2023 Nov 4;23:258. doi: 10.1186/s12874-023-02082-5 (PMC10625188; doi:10.1186/s12874-023-02082-5)

**Supplementary Information**

**Utilization of anonymization techniques to create an external control arm for clinical trial data**

Juha Mehtälä^1*^, Mehreen Ali^2,3^, Timo Miettinen^2,3^, Liisa Partanen^4^, Kaisa Laapas^4^, Petri T. Niemelä^4^, Igor Khorlo^5^, Sanna Ström^4^, Samu Kurki^4^, Jarno Vapalahti^4^, Khaled Abdelgawwad^5^, Jussi V. Leinonen^4^

^1^MedEngine Oy, Helsinki, Finland

^2^Veil.AI Oy, Helsinki, Finland

^3^Institute for Molecular Medicine Finland (FIMM), HiLIFE, University of Helsinki, Helsinki, Finland

^4^Bayer Oy, Espoo, Finland

^5^Bayer AG, Germany

*Corresponding author

Juha Mehtälä, PhD

+358 40 773 5059

juha.mehtala@medengine.fi

**Supplementary information**

*Selection of the anonymization framework*

The data authority that regulates the use of RWD in Finland (Findata) requires the use of *k*-anonymity (*k* = 5), and this was considered as the starting point for the selection of the anonymization framework. Moreover, two different options were considered. First, the (*k, ε*)-anonymity framework , which is a combination of *k*-anonymity on a set of quasi-identifiers and *ε*-differential privacy over the whole dataset. [1] This method overcomes the limitations of either of these approaches, such as the curse of dimensionality for *k*-anonymity and significant information loss caused by the *ε*-differential privacy mechanism. Second, the *ε*-safe *k*-anonymization approach, which offers robust (privacy-wise) results. [2]

The selection of the anonymization framework was conducted prior to the actual analyses in this study and was based on evaluation of the privacy of these above two candidate methods (Li et al. [2], and Holohan et al. [1]). In the evaluation, an anonymized and publicly available dataset (Framingham - heart disease dataset) was used. [3] The Framingham dataset is comparable in size (3,327 vs. 4,260 subjects) to the study dataset and contains similar variables. Both the two candidate methods were evaluated by performing membership inference (MI) attacks using the protocol described to the anonymized data. [4]

Based on the evaluation, Li et al. method may offer slight improvement against membership inference attacks compared to the selected method described by Holohan et al.. In addition, the theoretical advantage of the Li et al. approach is that it considers the differential privacy composability problem in the case of multiple data publications. However, since the Li et al. *ε*-safe *k*-anonymization approach relies on sampling, the data quality becomes more difficult to control, especially with imbalanced data. Therefore, the (*k, ε*)-anonymity framework by Holohan et al. was selected. [1,2]

*Selection of the anonymization parameters*

Unlike for *k*-anonymity (see above), there is currently no guidance or requirements from the data authority regarding the selection of the *ε*-parameter. Commonly used values of *ε* in the literature range from 0.01 to 7 depending on the use case and domain and it is difficult to

create generic rules for choosing the correct ε. [5] The driving factor for the selection of *ε* for this project was to ensure that anonymized real-world data had adequate privacy protections for it to be considered safe by the data authority (Findata). Meeting the privacy criteria defined by the data authority and assessing anonymity by the authority is required to export the results from the safe data environment. Data utility is a complex question and was therefore considered to be out of the scope in the selection of *ε.*

Recent works by Mehner et al. and He et al. suggest using privacy risk and privacy leak criteria for choosing the *ε* because these are more understandable for generic users. [6,7] Using the model proposed by Mehner et al., the maximum privacy risk for the study dataset for *ε* values 0.01 to 7 results in $\rho$ = 0.012% - 11.77% and for the chosen *ε* = 3.46 (ln 32), which lies in the middle of the range of recommended values, the maximum privacy risk $\rho$ is 0.39%. However, this value represents the worst-case, or maximum, privacy risk, and in practice, the actual post-anonymization privacy risk is likely to be lower for most subjects.

To validate the implementation and the privacy guarantees of the (*k, ε*)-anonymity framework across a range of k and ε values where *k* = 5, 10, 20, and *ε* = 1.39 (ln 4), …, 6.24 (ln 512) was performed using the Framingham dataset and (MI) attack approach. [4]

Based on the MI-attack model, which is different from the linking risk threat-model used by Holohan et al., support towards the safety claims of the (*k, ε*)-anonymity framework was found. [1] Finally, MI analysis on the anonymized study data was performed, to test the impact of the choice of *ε* = 3.46, and the results were found to be consistent with previous findings regarding the privacy assurances of (*k, ε*)-anonymity framework. The level of anonymization with the selected approach was also approved by the Findata authority.

### References

1. Holohan N, Antonatos S, Braghin S, Mac Aonghusa P. ($k$,$\epsilon$)-Anonymity: $k$-Anonymity with $\epsilon$-Differential Privacy [Internet]. 2017 [cited 2023 Aug 22];Available from: http://arxiv.org/abs/1710.01615

2. Li N, Qardaji W, Su D. On sampling, anonymization, and differential privacy or, k-anonymization meets differential privacy [Internet]. In: Proceedings of the 7th ACM Symposium on Information, Computer and Communications Security. New York, NY, USA: Association for Computing Machinery; 2012 [cited 2023 Feb 2]. page 32–3.Available from: https://doi.org/10.1145/2414456.2414474

3. Ashish Bhardwaj. Framingham heart study dataset [Internet]. [cited 2023 Aug 22];Available from: https://www.kaggle.com/dsv/3493583

4. El Emam K, Mosquera L, Fang X. Validating a membership disclosure metric for synthetic health data. JAMIA Open 2022;5:ooac083.

5. Hsu J, Gaboardi M, Haeberlen A, Khanna S, Narayan A, Pierce BC, et al. Differential Privacy: An Economic Method for Choosing Epsilon. In: 2014 IEEE 27th Computer Security Foundations Symposium. 2014. page 398–410.

6. Mehner L, Voigt SN von, Tschorsch F. Towards Explaining Epsilon: A Worst-Case Study of Differential Privacy Risks [Internet]. In: 2021 IEEE European Symposium on Security and Privacy Workshops (EuroS&PW). Vienna, Austria: IEEE; 2021 [cited 2023 Aug 22]. page 328–31.Available from: https://ieeexplore.ieee.org/document/9583708/

7. He X, Hong Y, Chen Y. Exploring the Privacy Bound for Diﬀerential Privacy: From Theory to Practice. EAI Endorsed Transactions on Security and Safety [Internet] 2019 [cited 2023 Aug 29];5. Available from: https://eudl.eu/doi/10.4108/eai.8-4-2019.157414

**Supplementary tables**

**Supplementary table 1.** Definitions of potential confounders.

| **Variable** | **Description** | **Specifications** | **Role in anonymization** |
| --- | --- | --- | --- |
| **Age** | Age at the index date (years) |  | Numerical variable |
| **Anemia** | Anemia; Hb < 130 g/dL for male, Hb < 120 g/dL for female, latest assessment before or at the index date; 1=yes / 0=no | If male and Hb < 130, or  if female and Hb < 120 | Categorical variable |
| **Anti-diabetic medication use** | Anti-diabetic medication use during baseline period: 1=yes / 0=no | ATC codes: A10 during baseline (1 year) | Categorical variable |
| **Anti-hypertensive medication use** | Anti-hypertensive medication use during the baseline period: 1=yes / 0=no | ATC codes: C02, C03, C07, C08, C09 during baseline (1 year) | Categorical variable |
| **Aortic arteriosclerosis** | Aortic arteriosclerosis during baseline period; 1=yes / 0=no | ICD-10 codes: I70.0 during baseline | Categorical variable |
| **Arterial hypertension** | Arterial hypertension during 5 years before cohort entry; 1=yes / 0=no | ICD-10 codes: I10, I11, I12, I13, I14, I15 or ICPC-2 codes: K86, K87 | Categorical variable |
| **Body mass index (BMI)** | BMI during 2 years before the index date; 1=>30 / 0=<30 |  | Quasi-identifier, categorical variable |
| **Carotid endarterectomy or stent** | Carotid endarterectomy or stent during baseline period; 1=yes / 0=no | Procedure code (NCSP) PAF14, PAQ14, PA6YT, PA7XT, PA7YT, or PA8YT, during baseline | Categorical variable |
| **Chronic heart failure** | Chronic heart failure during the baseline period; 1=yes / 0=no | ICD-10 codes: I50, I11.0, I13.0, I13.2 or  ICPC-2 code K77 during baseline | Categorical variable |
| **Chronic kidney disease** | Chronic kidney disease during baseline; 1=yes / 0=no | ICD-10 codes: N18, N19 or  eGFR <60ml/min/1.73m^2^, during baseline  (1 year) | Categorical variable |
| **Chronic obstructive pulmonary disease (COPD)** | COPD during baseline period: 1=yes / 0=no | ICD-10 codes: J43, J44 or  ICPC-2 code: R95 | Quasi-identifier, categorical variable |
| **Coronary artery disease** | Coronary artery disease during baseline period; 1=yes / 0=no | ICD-10 codes: I20, I21, I22, I23, I24, I25 or ICPC-2 codes: K74, K75, K76 | Categorical variable |
| **Diabetes mellitus** | Diabetes mellitus during baseline period; 1=yes / 0=no | ICD-10 codes: E10 or  ICPC-2 codes: T89, T90 during baseline | Categorical variable |
| **History of ISTH major bleeding** | History of ISTH major bleeding during the baseline period; 1=yes / 0=no | Applied definition of ISTH major bleed | Categorical variable |
| **History osteoporotic fracture** | History of hip fracture or osteoporosis during the baseline period; 1=yes / 0=no | ICD-10 codes: S72.0, M80, M81 | Categorical variable |
| **History of stroke** | History of stroke between days 30–365 during the baseline period; 1=yes / 0=no | ICD-10 codes: I61, I63, G46, or  ICPC-2 code: K90 | Categorical variable |
| **Hyperlipidemia** | Hyperlipidemia during the baseline period; 1=yes / 0=no | ICD-10 code: E78 or  ICPC-2 code: T93 | Categorical variable |
| **Hyperthyroidism** | Hyperthyroidism during baseline period: 1=yes / 0=no | ICD-10 codes: E05 | Categorical variable |
| **Hypothyroidism** | Hypothyroidism during baseline period: 1=yes / 0=no | ICD-10 codes: E01, E02, E03, E89.0, E89.1, E89.9 | Categorical variable |
| **Low body weight**  **(body weight < 60kg)** | Low body weight (body weight 1= <60kg / 0 = >=60kg) assessment closest to the index date |  | Quasi-identifier, categorical variable |
| **Malignancy** | Malignancy during baseline period; 1=yes / 0=no | ICD-10 codes C | Categorical variable |
| **Myocardial infarction** | Myocardial infarction during baseline period; 1=yes / 0=no | ICD-10 codes: I21, I22 or  ICPC-2 code: K75 | Categorical variable |
| **Non-steroidal anti-inflammatory drugs (NSAIDs)** | Prior or concomitant chronic use of non-steroidal anti-inflammatory drugs (NSAIDs) (including salicylic acid derivatives and excluding aspirin) during the baseline period; 1= yes / 0=no | ATC codes M01A | Categorical variable |
| **Percutaneous coronary intervention** | Percutaneous coronary intervention during baseline period; 1=yes/0=no | Procedure codes (NCSP): FN1, FN2, FN3 | Categorical variable |
| **Peripheral arterial disease** | Peripheral arterial disease during the baseline period; 1=yes / 0=no | ICD-10 code: I70.2  ICPC-2 codes: K92 | Categorical variable |
| **Platelet aggregation inhibitors** | Prior or concomitant use of platelet aggregation inhibitors (excluding heparin and aspirin) during the baseline period; 1=yes / 0=no | ATC codes B01AC (excluding B01AC06) | Categorical variable |
| **Prior or concomitant use of Histamine-2** | Prior or concomitant use of Histamine-2 receptor blocker; 1=yes / 0=no | ATC codes A02BA | Categorical variable |
| **Prior or concomitant use of (Selective Serotonin Reuptake Inhibitors) SSRIs** | Prior or concomitant use of SSRIs during the baseline period; 1=yes / 0=no | ATC codes N06AB | Categorical variable |
| **Prior use of heparins** | Prior use of heparins during baseline period; 1=yes / 0=no | ATC codes B01AB | Categorical variable |
| **Prior use of novel oral anticoagulants (NOACs)** | Prior use of NOACs during the baseline period;1=yes / 0=no | ATC codes B01AE, B01AF | Categorical variable |
| **Serum creatinine >= 1.5mg/dL** | Serum creatinine >=1.5mg/dL at the latest assessment before the index date; 1=yes / 0=no |  | Categorical variable |
| **Sex** | Male or female |  | Quasi-identifier, categorical variable |
| **Smoking status** | Smoking during the baseline period; 1=yes / 0=no | No includes former, never and missing | Quasi-identifier, categorical variable |
| **Transient ischemic attack (TIA)** | TIA during the baseline period; 1=yes / 0=no | ICD-10 code: G45 or  ICPC-2 code: K89 | Categorical variable |
| **Time in days since atrial fibrillation** | Time in days since the first atrial fibrillation diagnosis at the index date; <=30, 30<x<90, =>90 | ICD-10 code: I48 or  ICD-9 code: 4273A | Categorical variable |
| **Use of proton pump inhibitors** | Prior or concomitant use of proton pump during the baseline period; 1=yes / 0=no | ATC codes: A02BC | Categorical variable |

**Abbreviations:** ATC, Anatomical Therapeutic Chemical classification; BMI, body mass index; COPD, chronic obstructive pulmonary disease; eGFR, estimated glomerular filtration rate; Hb, hemoglobin; ICD-10, International Classification of Diseases 10^t^h Revision; ICPC-2, International Classification of Primary Care, 2nd edition; ISTH, International Society on Thrombosis and Haemostasis; NCSP, NOMESCO Classification of Surgical Procedures; NOAC, novel oral anticoagulant; NSAID, Non-steroidal anti-inflammatory drugs; SSRI, selective serotonin reuptake inhibitors; TIA, transient ischemic attack.

**Supplementary table 2.** Baseline summaries for the pseudonymized and anonymized real-world data sets and randomized controlled trial data set after propensity score matching, after PS-matching weighting, and after PS-overlap weighting.

| **Variable** | **Matched** | | | |  | **Weighted (MW)** | | | |  | **Weighted (OW)** | | | |
| --- | --- | --- | --- | --- | --- | --- | --- | --- | --- | --- | --- | --- | --- | --- |
|  | **RWD** | | **RCT** | |  | **RWD** | | **RCT** | |  | **RWD** | | **RCT** | |
|  | **Pseudonymized** | **Anonymized** | **Pseudonymized*** | **Anonymized*** |  | **Pseudonymized** | **Anonymized** | **Pseudonymized*** | **Anonymized*** |  | **Pseudonymized** | **Anonymized** | **Pseudonymized*** | **Anonymized*** |
| **n** | 223 | 226 | 223 | 226 |  | 219.55 | 221.68 | 221.11 | 222.24 |  | 173.71 | 174.92 | 173.71 | 174.92 |
| **Age, mean (SD)** | 75.71  (8.61) | 75.24  (9.55) | 74.93  (8.03) | 74.89 (8.00) |  | 75.28  (9.22) | 75.21 (10.18) | 74.96  (7.98) | 74.90  (8.00) |  | 75.34  (9.21) | 75.26 (10.16) | 75.34  (8.00) | 75.26  (8.03) |
| **Anemia, n (%)** | 31 (13.9) | 28 (12.4) | 26 (11.7) | 26 (11.5) |  | 26.8 (12.2) | 26.6 (12.0) | 25.3 (11.4) | 25.3 (11.4) |  | 21.8 (12.6) | 22.0 (12.6) | 21.8 (12.6) | 22.0 (12.6) |
| **Anti-diabetic medication use,**  **n (%)** | 68 (30.5) | 73 (32.3) | 68 (30.5) | 69 (30.5) |  | 65.7 (29.9) | 65.3 (29.4) | 66.0 (29.9) | 66.4 (29.9) |  | 50.8 (29.2) | 51.1 (29.2) | 50.8 (29.2) | 51.1 (29.2) |
| **Anti-hypertensive medication use,**  **n (%)** | 220 (98.7) | 222 (98.2) | 220 (98.7) | 223 (98.7) |  | 216.8 (98.7) | 218.9 (98.7) | 218.1 (98.6) | 219.2 (98.7) |  | 171.1 (98.5) | 172.3 (98.5) | 171.1 (98.5) | 172.3 (98.5) |
| **Aortic arteriosclerosis, n (%)** | <5 (<2.2) | <5 (<2.2) | <5 (<2.2) | <5 (<2.2) |  | <5 (<2.3) | <5 (<2.3) | <5 (<2.3) | <5 (<2.2) |  | <5 (<2.9) | <5 (<2.9) | <5 (<2.9) | <5 (<2.9) |
| **Arterial hypertension,**  **n (%)** | 189 (84.8) | 189 (83.6) | 193 (86.5) | 196 (86.7) |  | 190.6 (86.8) | 192.6 (86.9) | 191.9 (86.8) | 193.0 (86.8) |  | 147.7 (85.0) | 148.8 (85.1) | 147.7 (85.0) | 148.8 (85.1) |
| **BMI ≥30 kg/m^2^, n (%)** | 68 (30.5) | 72 (31.9) | 62 (27.8) | 65 (28.8) |  | 65.4 (29.8) | 66.6 (30.1) | 65.9 (29.8) | 66.9 (30.1) |  | 47.7 (27.4) | 48.5 (27.7) | 47.7 (27.4) | 48.5 (27.7) |
| **Carotid endarterectomy or stent, n (%)** | <5 (<2.2) | <5 (<2.2) | <5 (<2.2) | <5 (<2.2) |  | <5 (<2.3) | <5 (<2.3) | <5 (<2.3) | <5 (<2.2) |  | <5 (<2.9) | <5 (<2.9) | <5 (<2.9) | <5 (<2.9) |
| **Chronic heart failure, n (%)** | 106 (47.5) | 103 (45.6) | 95 (42.6) | 99 (43.8) |  | 98.3 (44.8) | 99.9 (45.1) | 96.9 (43.8) | 98.2 (44.2) |  | 72.6 (41.8) | 73.8 (42.2) | 72.6 (41.8) | 73.8 (42.2) |
| **Chronic kidney disease, n (%)** | 37 (16.6) | 27 (11.9) | 36 (16.1) | 37 (16.4) |  | 35.0 (15.9) | 34.9 (15.8) | 35.1 (15.9) | 35.3 (15.9) |  | 27.6 (15.9) | 27.8 (15.9) | 27.6 (15.9) | 27.8 (15.9) |
| **COPD, n (%)** | 24 (10.8) | 22 (9.7) | 20 (9.0) | 21 (9.3) |  | 20.3 (9.2) | 20.8 (9.4) | 20.4 (9.2) | 21.0 (9.5) |  | 15.2 (8.7) | 15.8 (9.0) | 15.2 (8.7) | 15.8 (9.0) |
| **Coronary artery disease, n (%)** | 53 (23.8) | 48 (21.2) | 40 (17.9) | 41 (18.1) |  | 43.6 (19.9) | 43.5 (19.6) | 40.6 (18.4) | 40.9 (18.4) |  | 33.5 (19.3) | 33.8 (19.3) | 33.5 (19.3) | 33.8 (19.3) |
| **Diabetes mellitus, n (%)** | 77 (34.5) | 91 (40.3) | 78 (35.0) | 79 (35.0) |  | 76.6 (34.9) | 76.5 (34.5) | 74.8 (33.8) | 75.2 (33.9) |  | 57.6 (33.2) | 58.0 (33.1) | 57.6 (33.2) | 58.0 (33.1) |
| **History of ISTH major bleeding, n (%)** | 17 (7.6) | 18 (8.0) | 20 (9.0) | 21 (9.3) |  | 18.5 (8.4) | 18.6 (8.4) | 18.5 (8.4) | 18.7 (8.4) |  | 13.9 (8.0) | 14.2 (8.1) | 13.9 (8.0) | 14.2 (8.1) |
| **History of osteoporotic fracture, n (%)** | 5 (2.2) | 5 (2.2) | 5 (2.2) | 5 (2.2) |  | <5 (<2.3) | <5 (<2.3) | 5.0 (2.3) | 5.0 (2.2) |  | <5 (<2.9) | <5 (<2.9) | <5 (<2.9) | <5 (<2.9) |
| **History of stroke, n (%)** | 16 (7.2) | 22 (9.7) | 17 (7.6) | 18 (8.0) |  | 19.0 (8.6) | 18.7 (8.4) | 17.9 (8.1) | 17.8 (8.0) |  | 15.0 (8.6) | 15.0 (8.6) | 15.0 (8.6) | 15.0 (8.6) |
| **Hyperlipidemia, n (%)** | 79 (35.4) | 86 (38.1) | 76 (34.1) | 78 (34.5) |  | 77.1 (35.1) | 76.9 (34.7) | 75.1 (34.0) | 75.6 (34.0) |  | 56.4 (32.5) | 56.8 (32.5) | 56.4 (32.5) | 56.8 (32.5) |
| **Hyperthyroidism, n (%)** | <5 (<2.2) | <5 (<2.2) | <5 (<2.2) | <5 (<2.2) |  | <5 (<2.3) | <5 (<2.3) | <5 (<2.3) | <5 (<2.2) |  | <5 (<2.9) | <5 (<2.9) | <5 (<2.9) | <5 (<2.9) |
| **Hypothyroidism, n (%)** | 24 (10.8) | 27 (11.9) | 24 (10.8) | 26 (11.5) |  | 24.6 (11.2) | 24.9 (11.3) | 26.0 (11.8) | 26.1 (11.7) |  | 20.2 (11.6) | 20.5 (11.7) | 20.2 (11.6) | 20.5 (11.7) |
| **Low body weight (body weight < 60kg), n (%)** | 194 (87.0) | 205 (90.7) | 197 (88.3) | 199 (88.1) |  | 194.7 (88.7) | 196.5 (88.6) | 196.6 (88.9) | 197.4 (88.8) |  | 154.4 (88.9) | 155.3 (88.8) | 154.4 (88.9) | 155.3 (88.8) |
| **Malignancy, n (%)** | 36 (16.1) | 42 (18.6) | 41 (18.4) | 41 (18.1) |  | 37.3 (17.0) | 38.0 (17.2) | 37.0 (16.8) | 37.5 (16.9) |  | 28.5 (16.4) | 28.9 (16.5) | 28.5 (16.4) | 28.9 (16.5) |
| **Myocardial infarction, n (%)** | 28 (12.6) | 24 (10.6) | 25 (11.2) | 25 (11.1) |  | 22.1 (10.1) | 22.2 (10.0) | 22.1 (10.0) | 22.4 (10.1) |  | 15.5 (8.9) | 15.8 (9.0) | 15.5 (8.9) | 15.8 (9.0) |
| **Non-steroidal anti-inflammatory drugs, n (%)** | 19 (8.5) | 16 (7.1) | 18 (8.1) | 18 (8.0) |  | 17.8 (8.1) | 17.9 (8.1) | 17.8 (8.0) | 17.9 (8.1) |  | 15.3 (8.8) | 15.5 (8.8) | 15.3 (8.8) | 15.5 (8.8) |
| **Percutaneous coronary intervention, n (%)** | 14 (6.3) | 14 (6.2) | 13 (5.8) | 13 (5.8) |  | 12.6 (5.7) | 12.5 (5.6) | 11.7 (5.3) | 11.7 (5.3) |  | 9.6 (5.5) | 9.6 (5.5) | 9.6 (5.5) | 9.6 (5.5) |
| **Peripheral arterial disease, n (%)** | 19 (8.5) | 18 (8.0) | 16 (7.2) | 15 (6.6) |  | 15.2 (6.9) | 15.4 (7.0) | 15.1 (6.8) | 15.3 (6.9) |  | 11.2 (6.4) | 11.4 (6.5) | 11.2 (6.4) | 11.4 (6.5) |
| **Platelet aggregation inhibitors, n (%)** | 202 (90.6) | 213 (94.2) | 209 (93.7) | 211 (93.4) |  | 204.7 (93.2) | 206.8 (93.3) | 206.5 (93.4) | 207.6 (93.4) |  | 161.6 (93.0) | 162.7 (93.0) | 161.6 (93.0) | 162.7 (93.0) |
| **Prior or concomitant use of Histamine-2, n (%)** | <5 (<2.2) | <5 (<2.2) | <5 (<2.2) | <5 (<2.2) |  | <5 (<2.3) | <5 (<2.3) | <5 (<2.3) | <5 (<2.2) |  | <5 (<2.9) | <5 (<2.9) | <5 (<2.9) | <5 (<2.9) |
| **Prior or concomitant use of SSRIs, n (%)** | 9 (4.0) | 7 (3.1) | 6 (2.7) | 6 (2.7) |  | 5.8 (2.6) | 5.9 (2.6) | 6.0 (2.7) | 6.0 (2.7) |  | <5 (<2.9) | <5 (<2.9) | <5 (<2.9) | <5 (<2.9) |
| **Prior use of heparins, n (%)** | 50 (22.4) | 53 (23.5) | 49 (22.0) | 50 (22.1) |  | 52.6 (24.0) | 52.5 (23.7) | 52.1 (23.5) | 52.3 (23.5) |  | 41.8 (24.1) | 42.1 (24.1) | 41.8 (24.1) | 42.1 (24.1) |
| **Prior use of NOACs, n (%)** | 126 (56.5) | 129 (57.1) | 132 (59.2) | 133 (58.8) |  | 123.3 (56.2) | 124.1 (56.0) | 126.1 (57.0) | 126.5 (56.9) |  | 98.5 (56.7) | 99.2 (56.7) | 98.5 (56.7) | 99.2 (56.7) |
| **Serum creatinine ≥ 1.5mg/dL,**  **n (%)** | 25 (11.2) | 22 (9.7) | 25 (11.2) | 26 (11.5) |  | 23.3 (10.6) | 23.3 (10.5) | 23.8 (10.8) | 24.2 (10.9) |  | 18.6 (10.7) | 18.9 (10.8) | 18.6 (10.7) | 18.9 (10.8) |
| **Sex = Male,**  **n (%)** | 126 (56.5) | 123 (54.4) | 128 (57.4) | 128 (56.6) |  | 122.2 (55.7) | 122.8 (55.4) | 122.0 (55.2) | 122.2 (55.0) |  | 95.3 (54.8) | 95.6 (54.7) | 95.3 (54.8) | 95.6 (54.7) |
| **Smoking status, n (%)** | 8 (3.6) | 15 (6.6) | 8 (3.6) | 9 (4.0) |  | 8.8 (4.0) | 8.9 (4.0) | 9.1 (4.1) | 9.3 (4.2) |  | 7.7 (4.5) | 8.0 (4.5) | 7.7 (4.5) | 8.0 (4.5) |
| **TIA, n (%)** | 11 (4.9) | 11 (4.9) | 10 (4.5) | 9 (4.0) |  | 10.2 (4.6) | 10.1 (4.6) | 10.6 (4.8) | 10.8 (4.8) |  | 7.8 (4.5) | 7.9 (4.5) | 7.8 (4.5) | 7.9 (4.5) |
| **Time in days since atrial fibrillation, n (%)** | | | | | | | | | | | | | | |
| *≤ 30* | 89 (39.9) | 92 (40.7) | 88 (39.5) | 89 (39.4) |  | 87.6 (39.9) | 89.0 (40.1) | 88.2 (39.9) | 88.5 (39.8) |  | 65.9 (38.0) | 66.2 (37.9) | 65.9 (38.0) | 66.2 (37.9) |
| *≥ 90* | 120 (53.8) | 121 (53.5) | 121 (54.3) | 123 (54.4) |  | 118.7 (54.1) | 119.4 (53.9) | 120.4 (54.5) | 121.2 (54.5) |  | 97.9 (56.3) | 98.7 (56.4) | 97.9 (56.3) | 98.7 (56.4) |
| *30<x<90* | 14 (6.3) | 13 (5.8) | 14 (6.3) | 14 (6.2) |  | 13.2 (6.0) | 13.3 (6.0) | 12.5 (5.7) | 12.5 (5.6) |  | 9.9 (5.7) | 10.0 (5.7) | 9.9 (5.7) | 10.0 (5.7) |
| **Use of proton pump inhibitors, n (%)** | 95 (42.6) | 106 (46.9) | 97 (43.5) | 100 (44.2) |  | 93.0 (42.3) | 93.2 (42.0) | 93.6 (42.3) | 93.8 (42.2) |  | 73.0 (42.0) | 73.5 (42.0) | 73.0 (42.0) | 73.5 (42.0) |

*Randomized controlled trial (RCT) data were not anonymized, and the results instead refer to matching/weighting with either pseudonymized or anonymized real-world data (RWD). **Abbreviations:** BMI, body mass index; COPD, chronic obstructive pulmonary disease; ISTH, International Society on Thrombosis and Haemostasis; MW, matching weighting; NOAC, novel oral anticoagulant; OW, overlap weighting; PS, propensity score; RCT, randomized controlled trials, RWD, real-world data; SD, standard deviation; SSRI, selective serotonin reuptake inhibitor; TIA, transient ischemic attack.

**Supplementary Figure**

**Supplementary figure 1.** Logistic regression model results (propensity-score model effects) for the confounders in anonymized and pseudonymized real-world data sets.


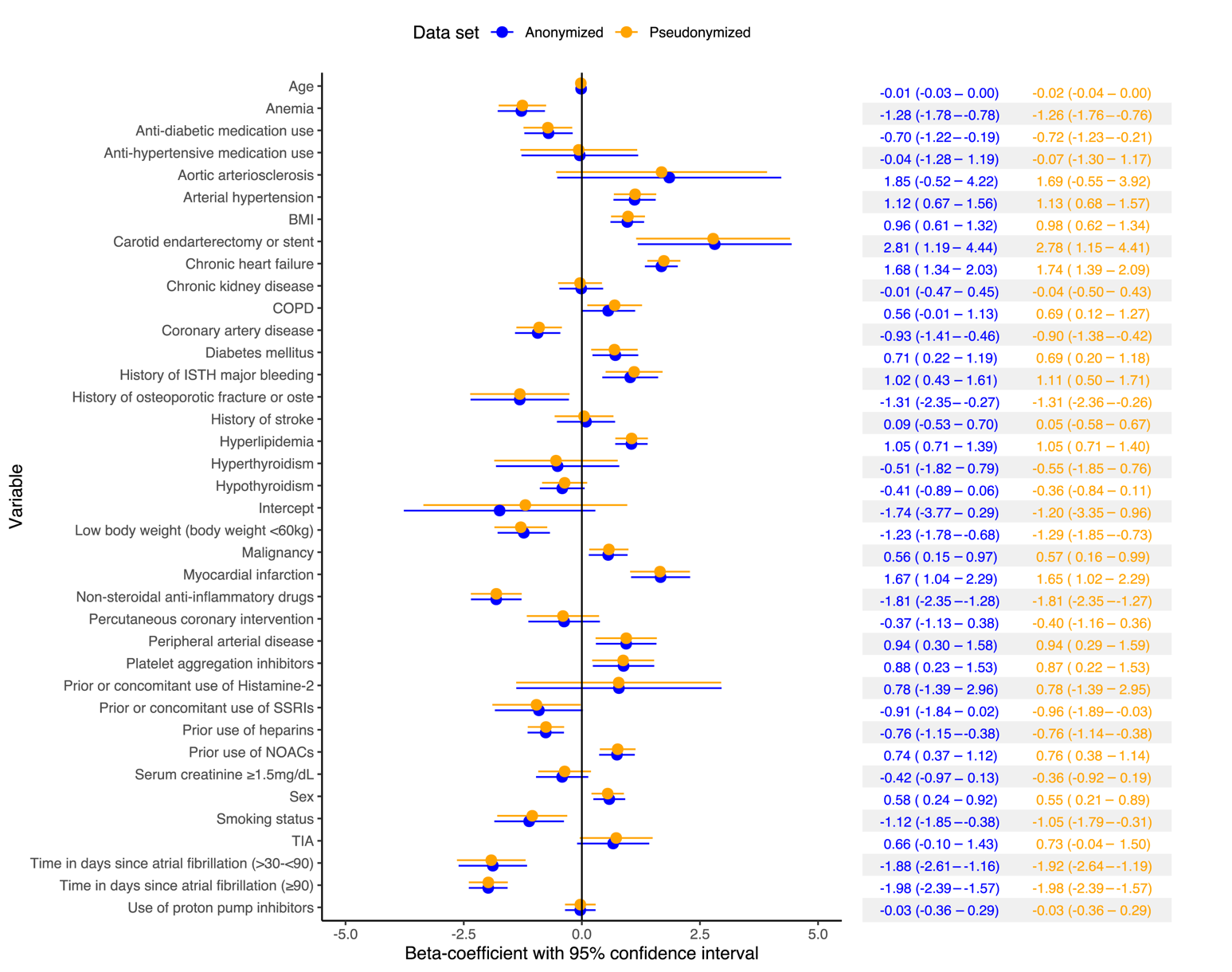

Supplement: Supplementary file 1 — Additional file 1: Table 1. Definitions of potential confounders. Table 2. Baseline summaries for the pseudonymized and anonymized real-world data sets and randomized controlled trial data set after propensity score matching, after PS-matching weighting, and after PS-overlap weighting. Figure 1. Logistic regression model results (propensity-score model effects) for the confounders in anonymized and pseudonymized real-world data sets. [file 12874_2023_2082_MOESM1_ESM.docx]
